# The introduction of a highly virulent PRRSV strain in pig farms is associated with a change in the pattern of influenza A virus infection in nurseries

**DOI:** 10.1186/s13567-024-01406-7

**Published:** 2024-11-09

**Authors:** Ivan Domingo-Carreño, Maria Soledad Serena, Gerard Eduard Martín-Valls, Hepzibar Clilverd, Laia Aguirre, Martí Cortey, Enric Mateu

**Affiliations:** Department of Animal Health and Anatomy, Universitat Autònoma de Barcelona, Travessera Dels Turons S/N, 08193 Cerdanyola del Vallès, Spain

**Keywords:** Influenza A virus, porcine reproductive and respiratory syndrome virus, maternally derived antibodies, coinfection

## Abstract

The present study aimed to determine the dynamics of influenza A virus (IAV) infection in two endemically infected farms (F1 and F2), where a longitudinal follow-up of piglets was performed from birth to 8–12 weeks of age. During the study, a highly virulent isolate of porcine reproductive and respiratory syndrome virus (PRRSV) was introduced on both farms. This allowed us to examine the impact of such introduction on the patterns of infection, disease, and the antibody response of pigs to IAV infection. The introduction of the new PRRSV strain coincided with a change in the dynamics of IAV infection on both farms. In F1, the cumulative incidence of IAV increased from 20% before the outbreak to 67.5%, together with the existence of animals that tested positive for IAV (RT‒qPCR) in nasal swabs for two or more consecutive samples. In F2, the cumulative incidence of IAV increased from 50% before the PRRSV outbreak to 70%, and the proportion of prolonged IAV shedders increased sharply. Additionally, some animals were infected with the same IAV twice during the observation period. In contrast to previous reports, our study revealed that prolonged shedding was not related to the titres of maternally derived antibodies at the time of infection but was significantly (*p* < 0.05) related to PRRSV infection status. Notably, both before and after the PRRSV outbreak, a high proportion of IAV-infected piglets did not seroconvert, which was significantly (*p* < 0.05) related to the hemagglutination inhibition titres against IAV when infected.

**Supplementary Information:**

The online version contains supplementary material available at 10.1186/s13567-024-01406-7.

## Introduction

Influenza A virus (IAV) is one of the most significant respiratory pathogens of pigs and is an important participant in the so-called porcine respiratory disease complex (PRDC), which results in significantly increased mortalities and economic losses for the swine industry annually.

The virus primarily infects the epithelial airway, causing the death of infected cells and an intense inflammatory response. In pigs, the hallmark lesions are necrotizing bronchitis and bronchiolitis, which, in severe cases, may involve the alveoli in a broncho-interstitial pneumonia pattern [1]. Additionally, epithelial denudation of the airway, together with the inflammatory response, facilitates the invasion of bacterial pathogens that can produce complicating infections [2].

Pigs play an important role in the epidemiology of IAV. The species has been postulated to be a “mixing vessel”, where mammalian and avian influenza viruses may converge. This is because pigs have both α-2,3 and α-2,6 sialic acid receptors [3, 4], making them susceptible to both types of IAV. The introduction of avian strains in pigs has been documented [5, 6], and at present, one of the predominant H1 lineages of Eurasian swine IAV (swIAV), H1C, has an avian origin [7].

The important role of pigs in IAV epidemiology became even more evident in 2009 with the emergence of the last pandemic IAV, an H1N1 that was the result of several reassortments involving three swIAV lineages: an H3N2 swine virus of North American origin (PB2, PB1, PA, NP, and NS segments), an H1 from the classical swine H1N1 lineage present in North America (HA), and an avian-like Eurasian swine lineage (NA, MP) [8, 9].

The epidemiology of swine influenza has not been fully elucidated. Classically, the disease is reported as acute respiratory disease outbreaks. However, in recent years, it has become increasingly evident that in most farms, the infection establishes an endemic cycle in which viral circulation can cause recurrent respiratory disease in nurseries or even in farrowing units [10–13]. The drivers of those endemic situations are poorly known and may depend on specific factors of each herd, such as the structure of the farm. However, some variables are often related to endemic status, including animal density and the introduction of susceptible animals, particularly gilts [14, 15].

One of the elements often cited as important drivers of IAV infection dynamics in endemic farms is maternally derived antibodies (MDA). It is known that homologous MDA can protect piglets [16]. However, under field conditions, not all sows have the same level of immunity or the same record of contact with the virus (e.g., with different viral strains). As a result, the amount and quality of MDA can vary widely within a herd. Moreover, several papers have shown that animals exposed to swIAV in the presence of MDA may experience some level of clinical protection but can still be infected [10, 11, 17]. It has been suggested that MDA may hamper the development of active immunity in infected pigs [18] or even result in the enhancement of respiratory disease upon heterologous challenge [19]. Additionally, the presence of MDA has been related to unusually prolonged shedding patterns in piglets [17, 20, 21].

In most pig farms, animals are infected not only by swIAV but also by a variety of viral and bacterial agents, contributing to the PRDC. While interactions between IAV and bacterial agents are most often explained by IAV’s ability to facilitate complicating bacterial infections [22, 23], interactions with other viruses have been less studied. In the case of swIAV, coinfection with other viruses, such as porcine reproductive and respiratory syndrome virus (PRRSV), has been shown to result in impaired replication of PRRSV but also in increased severity of the disease [24].

In a recent study, Martin-Valls et al. [25] reported the introduction of a highly virulent PRRSV strain in Spain. Highly virulent PRRSV isolates have high potential for dysregulating pigs’ immune response [26, 27]. The aim of the present study was to assess the dynamics of swIAV infection and the humoral response of pigs on endemically infected farms and to examine the impact of the introduction of a highly virulent PRRSV-1 strain on the patterns of swIAV infection, disease, and serological response.

## Materials and methods

### Farms

Longitudinal studies (two per farm) were conducted on two selected farrow-to-fattening farms (identified as F1 and F2), allocating animals from birth to 9 weeks of age (woa). Both farms were located in the central Catalonia region, a medium pig density area in Spain.

F1 was a 900-sow operation with biweekly batches (approximately 80 sows/batch). The farm was known to be positive for IAV since 2018, but no record of the circulating subtype and lineage was available. According to previous monitoring data, the farm was positive but stable for PRRSV, with sows being vaccinated with a modified live vaccine (category II-vx according to the scheme proposed by Holtkamp et al. [28]). The farm did not vaccinate against IAV. F1 experienced recurrent respiratory disease (sneezes and cough), which was usually noticed after the 5^th^-6^th^ woa. Mortality in nurseries reached 5% in 2020, the year before the follow-up, mostly due to respiratory disease. For F1, the first longitudinal study (L1) was carried out from October to December 2021, whereas the second longitudinal study (L2) was conducted between May and July 2022.

F2 was a 1400-sow operation working with weekly batches (approximately 62 sows/batch). This farm has been positive for swIAV since 2018, but no information about the subtype or lineage was available. F2 was positive for PRRSV, with occasional detection of PCR-positive animals at weaning (category I-B-positive unstable, low prevalence, according to Holtkamp’s scheme [28]), and sows were vaccinated every 4 months with a modified live PRRSV vaccine. The farm did not vaccinate against IAV. Similar to F1, the nursery at F2 suffered from recurrent respiratory disease starting at 5–6^th^ woa, with a mortality rate in nurseries of 5.3% in 2020. For F2, L1 was carried out from October to December 2021, whereas L2 was carried out from February to April 2022.

On both farms, piglets were weaned at 28 days of age and stayed in the nursery until the 9^th^ woa, when they were moved to a fattening unit. Both farms purchased gilts from the same source. Replacements were purchased as PRRSV- and IAV-negative (6 times a year and tested during the quarantine period), and they were allocated to a quarantine facility that was > 4 km away from any other pig farm. Gilts remained there for at least two months and were vaccinated against PRRSV-1 with a modified live vaccine, porcine circovirus type 2 (PCV2), and *Mycoplasma hyopneumoniae*.

### Sample collection and clinical evaluation

For each herd and longitudinal study, 40 piglets from 10 different litters were randomly selected. For this purpose, 10 sows were randomly selected from a farrowing batch. Piglets were ear-tagged individually at birth and were followed from the 1^st^ until the 8^th^ woa for L1, whereas in L2, pigs were followed until the 12^th^ woa. Nasal swab and serum samples were collected from pigs at 1, 3, 5, and 8 woa for study L1. For the L2 study, the same type of samples were collected at 1, 3, 5, 6, 7, 8, and 12 woa for F1, whereas for F2, pigs were sampled at those timepoints plus at 4 woa. At each sampling point, mortality rates and respiratory scores were recorded.

For respiratory scores, the researchers ensured that all the animals were awake and active by making noise and clapping. Then, the researchers waited for 1 min before starting to record sneezing (S), coughing (C), and deep coughing (DC) for 2 min. This procedure was repeated 3 times, and the results were calculated by dividing the total number of S, C, and DC by the number of animals and minutes.

### RT-qPCR analysis

RNA extraction from nasal samples was performed via the MagMax CORE nucleic acid purification kit and a KingFisher robot (Thermo Fisher, Madrid, Spain). For initial virus detection, RT-qPCR targeted to the IAV matrix gene was performed following the protocol described by Busquets et al. [29], employing AgPath-ID™ One Step RT-PCR reagents (Thermo Fisher). Samples yielding C_t_ values ≤ 37.0 were classified as positive, whereas results between 37.1 and 39.9 were considered inconclusive and repeated. If the result remained ≥ 37.1 upon repetition, it was considered negative. The samples were also examined for PRRSV using a commercial kit (VETMAX™ PRRSV EU & NA 2.0 Kit, Thermo Fisher) according to the manufacturer’s instructions. For the purpose of the present study, and on the basis of the RT-qPCR data, animals that tested positive were classified into three categories: a) “new cases”; namely, the first detection of the virus for that animal; b) “prolonged shedders”; that is, animals that tested positive in two or more consecutive samplings; and c) “repeated infections”; namely, animals that tested positive after testing negative in non-consecutive sampling.

### swIAV and PRRSV isolation, sequencing, and phylogenetic analyses

Positive samples yielding C_t_ values < 31 for swIAV were selected for virus isolation in Madin–Darby canine kidney (MDCK) cells (ATCC CRL-2936™), as previously described [30]. To confirm virus isolation, supernatants from cell cultures displaying cytopathic effects were tested by RT-qPCR, and the cell cultures were stained with a specific blend of monoclonal antibodies against IAV (clones A1 and A3; Merck, Spain), along with a fluorescent anti-mouse IgG secondary antibody (Merck). For PRRSV, virus isolation was performed by inoculating porcine alveolar macrophages (PAMs) with sera from RT‒qPCR PRRSV-positive animals. Isolation was confirmed by staining inoculated PAMs with a specific monoclonal antibody against PRRSV (clone 1CH5, God Standard Diagnostic, Madrid, Spain) and a fluorescent anti-mouse IgG secondary antibody (Merck).

RNA was extracted from the swIAV and PRRSV isolates using TRIzol^®^ LS Reagent (Thermo Fisher) according to the manufacturer’s protocol. The extracted RNA was used to sequence the whole virus genomes using an Illumina® MiSeq platform at the Genomics Service of UAB. The output reads, in fastaq format (doubled pairs), were checked for quality using Trimmomatic (matching of forward and reverse sequences and quality index > 20). High-quality reads were then filtered using PRRSV or swIAV sequence references. For swIAV, the reference sequences encompassed all known porcine lineages for each of the eight genome segments. Genome consensuses were generated using the simple consensus marker tool [31]. The segments were subsequently manually aligned and trimmed using the BioEdit sequence alignment editor for Windows [32]. The resulting consensus sequence was blasted against available sequences in GenBank.

The obtained swIAV sequences were phylogenetically compared (Bayesian analysis using Beast [33]) with sets of contemporary Eurasian sequences to ascertain the genetic relatedness of the strains detected at each farm. Hemagglutinin was classified following the criteria outlined by Anderson et al. [34]. Genotype classification was determined using the scheme established by Watson et al. [35]. For PRRSV, a set of local Spanish sequences was used for phylogenetic comparison and analysed as described above.

### Serological assays

Specific antibodies against swIAV were initially determined using a commercial competition ELISA (Swine Influenza Virus Ab, IDEXX Laboratories, Spain). The results are expressed according to the manufacturer’s directions as the ratio of the optical density (OD) of each tested sample to the OD of a negative control provided by the kit, referred to as the sample-to-negative sample (S/N ratio). The cut-off was set at 0.6; accordingly, sera yielding optical densities < 0.6 were positive. Sera were subsequently examined by the hemagglutinin inhibition (HI) test. Serum samples were tested in duplicate, and the assay was performed with 4 hemagglutinating units (HAUs) per well, following the protocol of the World Organization for Animal Health published in the Manual of Diagnostic Tests and Vaccines for Terrestrial Animals [36]. This test employed turkey erythrocytes and the virus circulating in the farm as the antigen.

A selection of samples (at least one piglet per sow) was also subjected to a virus neutralization test (VNT). Sera were first treated at 56 °C for 30 min and mixed with a suspension containing 2000 TCID50/mL TPCK trypsin-treated swIAV (the resident isolate for each farm was used). MDCK-cell monolayers in 96-well plates were inoculated with the mixture and incubated at 37 °C for 48 h in 5% CO_2_. The plates were subsequently incubated with a blend of anti-influenza A monoclonal antibodies specific for the nucleoprotein antigen (Merck, Spain), and the test was revealed by adding an anti-mouse Ig-FITC (Merck). The average number of fluorescent foci obtained with sera devoid of anti-influenza antibodies was determined in previous experiments. The plates were examined under a fluorescence microscope.

### Statistical analyses

The statistical analyses were performed using GraphPad Prism 10.0.3.275 and R version 4.2.1. Statistical significance was set at *p* < 0.05. Chi-square tests were used to compare proportions across different groups, whereas Mann‒Whitney or Kruskal‒Wallis tests were used for the comparison of numeric data between groups. Linear regression and correlation analyses were employed to relate C_t_ values with HI or VNT titres.

## Results

### Dynamics of swIAV and PRRSV infections, mortality rates, and clinical observations

#### Farm 1

**Figure 1:**
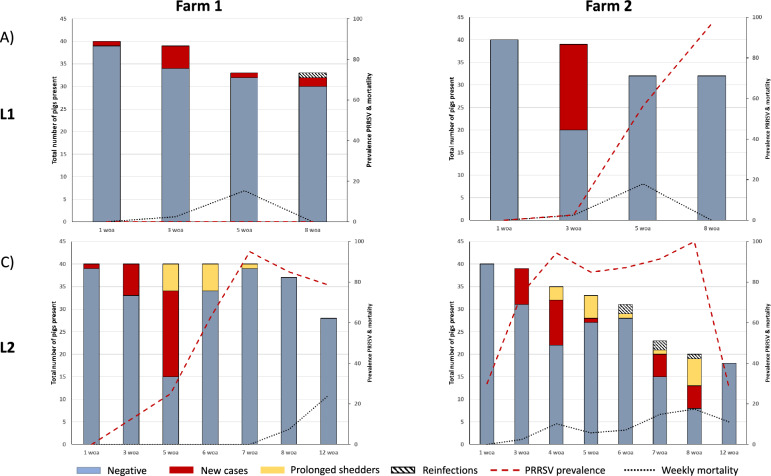
**Dynamics of swIAV and PRRSV infection in the studied farms.** Weekly mortality rates for each sampling period and farm. Left: Farm 1, Right: Farm 2; upper: longitudinal study 1 (L1), lower: longitudinal study 2 (L2). Red bars indicate the number of animals that tested positive for swIAV in nasal swabs for the first time; yellow bars indicate the number of animals that tested positive for swIAV for two or more consecutive samplings; dashed bars indicate the number of animals that tested positive for swIAV after being negative; and grey bars indicate the animals that tested negative for swIAV. The dotted red lines show the proportion of PRRSV-viremic animals at each timepoint. The black dotted line shows the mortality rate between two consecutive periods.

Figure 1 shows the infection dynamics as determined by RT‒qPCR analysis of nasal swabs. In L1 of F1, the cumulative incidence of swIAV was 20.0% (8/40; CI_95%_ = 9.6–36.1%). Positive animals were identified as early as the first woa (1/40; 2.5%; CI_95%_ = 0.1–14.7%), with further detections at 3 woa (5/39; 12.8%; CI_95%_ = 4.8–28.2%), 5 woa (1/33; 3.0%; CI_95%_ = 0.2–17.5%), and 8 woa (3/33; CI_95%_ = 2.4–25.5%). One of the animals detected as swIAV-positive at 8 woa had already tested positive at 3 woa. No PRRSV-positive piglets were found in this first longitudinal study, confirming that the farm was stable and that PRRSV-positive piglets were not weaned. Throughout the study period, a total of 7 animals died (17.5%; CI_95%_ = 7.9–33.4%), with 6 deaths occurring between the 3^rd^ and the 5^th^ woa; however, none tested positive for swIAV in the last sampling before death.

In L2, the cumulative incidence was 67.5% (27/40; CI_95%_ = 50.8–80.9%), which was significantly higher than that in L1 (*p* < 0.001; relative risk = 3.9, CI_95%_ = 2.0–7.9). Once again, swIAV was detected from the first woa (1/40, 2.5%, CI_95%_ = 0.1–14.7%), with subsequent detections at 3 woa (7/40; 17.5%; CI_95%_ = 0.1–14.7%), 5 woa (25/40; 62.5%, CI_95%_ = 45.8–76.3%), 6 woa (6/40; 15%; CI_95%_ = 0.1–14.7%; 6.3–30.5%), and 7 woa (1/37; 2.7%; CI_95%_ = 0.1–15.8%). No swIAV-positive animals were detected beyond the 7^th^ woa. Notably, all pigs that tested positive for swIAV at 6 or 7 woa were positive at least one week before or more (see Additional file 1). Additional file 2 shows the distribution of C_t_ values for swIAV-positive samples from both farms. In F1, 11 isolates from both longitudinal follow-ups were subjected to whole-genome sequencing. In all the cases, the same swIAV strain was detected, belonging to the 1B.1.2.1 clade according to Anderson’s classification [34].

With respect to PRRSV, just prior to the commencement of the L2 study, the farm became infected by a highly virulent isolate belonging to the new clade described by Martin-Valls et al. [25] (with 97.7% identity to the first described isolate of this clade, R1, GenBank accession number OM893828). At the beginning of the sampling, PRRSV was not detected in newborns, but by the age of 3 weeks, 12.5% of the piglets were already viremic, and this proportion increased to 95% by 7 woa.

In L1, no respiratory disease was noted until the 3^rd^ woa, when sneezing animals were first detected. Sneezing persisted until the 8^th^ woa. Coughing did not appear until the 5^th^ woa and increased by the 8^th^ woa. In L2, sneezing and coughing were detected from 3 woa onwards. Mortality was nil until the 7^th^ woa, after which 13 animals died until the end of the observation at 12 woa (13/40; 32.5% CI_95%_ = 19.1–49.2%). All dead animals had been infected by PRRSV, with only one of them testing positive for swIAV during the last sampling before death.

#### Farm 2

For F2, in L1, the cumulative incidence of swIAV was 50% (20/40; CI_95%_:34.1–65.9%), similar to that of F1. No swIAV-positive animals were detected until the 3^rd^ woa (19/39; 48.7%; CI_95%_:32.7–65.0%). Afterwards, none of the animals tested positive. In L2, the cumulative incidence increased to 70.0% (28/40; CI_95%_:53.3–82.9%, p = 0.06). In this case, swIAV was again detected for the first time at 3 woa (8/39; 20.5%; CI_95%_: 9.9–36.9%), followed by 4 woa (13/36; 36.1%; CI_95%_:21.5–53.8%), 5 woa (6/35; 17.1%; CI_95%_:7.2–34.3%), 6 woa (3/32; 9.4%; CI_95%_:2.5–26.2%), 7 woa (8/25; 32%; CI_95%_:15.7–53.6%), and 8 woa (12/24; 50%; CI_95%_:29.7–70.4%).

Notably, a considerable proportion of the positive samples corresponded to animals detected as shedders in two or more consecutive samplings (16/28 swIAV-positive individuals, 57.1% CI_95_: 37.4–75.0%). Additionally, five animals (17.9% of the infected pigs) were identified as reinfected by the same swIAV isolate after testing negative for one or more weeks.

In F2, 20 isolates corresponding to both longitudinal follow-ups were full-genome sequenced. In this case, all swIAV isolates recovered from F2 had the same genotype, belonging to the 1C.1.2.1 clade according to Anderson’s classification [34].

With respect to PRRSV, the farm was infected with different L1 and L2 isolates, as determined by full-genome sequencing. While in L1, the resident PRRSV strain belonged to a cluster of classical PRRSV-1.1 isolates, in L2, the farm was infected by a highly virulent strain corresponding to the recently reported clade. In L1, PRRSV was first detected at 3 woa (2.6%), and the prevalence of infected animals increased in the nurseries to 97% at 8 woa. In L2, PRRSV infection was already detected at birth in 30% of the newborns, increasing to 74% by 3 woa and oscillating between 85 and 100% of viremic animals by the 8^th^ woa. At 12 woa, 28% of the present animals were still infected with PRRSV (Figure 1).

In L1, respiratory signs were not detected until the 5^th^ woa and persisted until the end of the nursery period. Overall mortality reached 20% (7/40; 17.5%; CI_95%_ = 7.9–33.4%) from birth to the 8^th^ woa, occurring all between the 3^rd^ and the 5^th^ woa. Five of these animals tested positive for IAV during the last sampling before death, whereas none of them tested positive for PRRSV before death. In contrast, in L2, coughing and deep coughing were already detected in one-week-old piglets and persisted until the end of the study. The overall mortality rate for L2 patients was 55% (22/40 CI_95%_ = 38.7–70.4%), with weekly mortality rates reaching 17%. Sixty-eight percent of the dead animals (15/22) were PRRSV positive in the last sampling immediately before death, of which 4 were coinfected by swIAV. Notably, the age at which pigs were first detected as positive for PRRSV had a strong impact on mortality in L2 pigs. Thus, animals infected at birth had a mortality of 83.3% (10/12) compared with 47% for animals infected at 3 woa (9/19) and 0% for animals infected at 4 woa (0/4) (*p* < 0.01). Table 1 summarizes the clinical records for the following farms.

**Table 1:** **Sneezing and cough indexes as recorded at 1, 3, 5, and 8 weeks of age**

		Weeks of age (n° animals)	Sneezing	Cough	Deep cough
FARM 1		1 (*n* = 144)	0	0	0
		3 (*n* = 140)	0.079	0	0
	L1	5 (*n* = 262)	0.047	0.007	0
		8 (*n* = 202)	0.107	0.111	0.004
		1 (*n* = 222)	0	0	0
		3 (*n* = 210)	0.009	0.001	0
	L2	5 (*n* = 260)	0.003	0.005	0
		8 (*n* = 240)	0.050	0.001	0
FARM 2		1 (*n* = 460)	0	0	0
		3 (*n* = 460)	0.072	0	0
	L1	5 (*n* = 261)	0.085	0.001	0.002
		8 (*n* = 254)	0.070	0.080	0.001
		1 (*n* = 429)	0.115	0.32	0.004
		3 (*n* = 268)	0.080	0.016	0.007
	L2	5 (*n* = 253)	0.077	0.013	0.003
		8 (*n* = 190)	0.137	0.017	0.009

L1, longitudinal study 1; L2, longitudinal study 2.

Each index was calculated as the average count of sneezes or coughs recorded in 1 min divided by the number of pigs present.

Taking advantage of the nasal swabs collected from the studied animals, it was possible to determine the shedding period for the highly virulent PRRSV circulating on both farms during L2. The calculation considered the first and the last sampling at which nasal swabs tested positive for PRRSV in the RT‒qPCR assay. The average shedding period for F1 L2 was 4.4 ± 2.2 weeks (range, 1–9 weeks), and for F2 L2, it was 5.3 ± 2.5 weeks (range, 2–11 weeks), with no significant differences between them. The individual data of nasal shedding of PRRSV for the batches where circulation of the virus was detected are available in Additional file 1. Interestingly, in one F1 L2 (n°126) animal and two F2 L2 (n° 112 and 113) animals that were infected by swIAV when they were already infected by PRRSV, PRRSV viremia was not detectable for one or more weeks while they were infected by swIAV but then resumed. All three animals experienced PRRSV nasal shedding during that period.

### Serology

**Figure 2:**
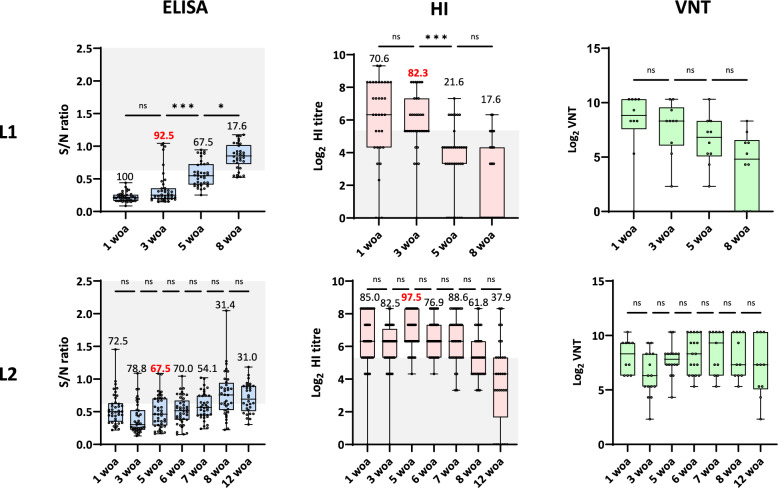
**Results of the serological analyses for swIAV performed at Farm 1 and longitudinal studies 1 and 2.** L1 = longitudinal study 1; L2 = longitudinal study 2. From left to right, the results obtained by ELISA, HI, and VNT are shown. The box and whisker plots indicate the minimum, maximum, median, and 25% and 75% quartiles. Each dot represents an individual result. For the ELISA and HI plots, the grey area delimits the threshold for positive results (S/N < 0.6 for ELISA) or a predicted correlate of protection (1:40 in HI). The number over the box indicates the proportion of examined individuals over the threshold, with the number in red corresponding to the proportion of seropositive animals at the moment when swIAV incidence was the highest for that batch of pigs. For VNT, no threshold is indicated, as no standard threshold has been determined for pigs. The average S/N values or titres were compared between consecutive weeks and are indicated in the graphs. *p < 0.05; **p < 0.01; ***p < 0.001; ****p < 0.0001; n.s. = nonsignificant difference.

In F1, most of the 1-woa animals had MDA in both L1 and L2. The HI test results were used as a reference, and a cut-off of 1:40 (5.3 log_2_) was used as an indication of potential protection against infection; at 1 woa, 70.6% of the piglets in L1 and 85.0% in L2 had relevant levels of antibodies. In L1, the proportion of HI-positive animals above the cut-off decreased in consecutive samplings, reaching 17.6% at 8 woa. In contrast, in L2, at 8 woa, the proportion of pigs with HI titres above 5.3 log_2_ (61.8%) was significantly greater than the values observed at the same age in L1 (*p* < 0.001); however, at 12 woa, this proportion significantly decreased to 37.9% (*p* < 0.05). The VNT results showed the same trend, with a clear decline in titre with age in L1, whereas in L2, the average titre and the proportion of positive pigs were greater throughout the study period (Figure 2).

**Figure 3:**
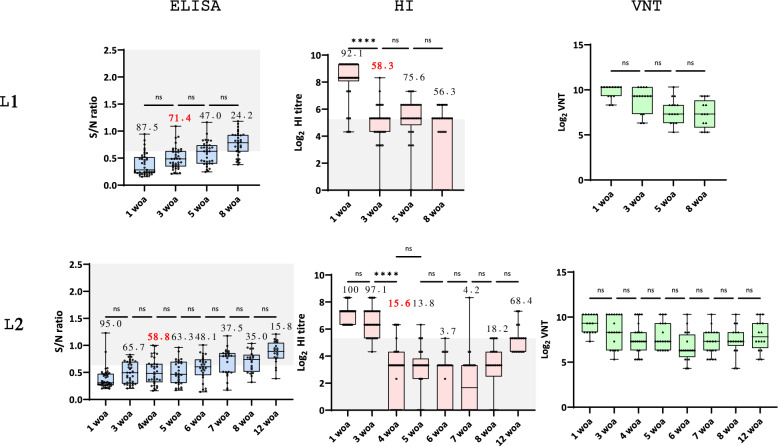
**Results of the serological analyses for swIAV performed at Farm 2 and longitudinal studies 1 and 2.** L1 = longitudinal study 1; L2 = longitudinal study 2. From left to right: ELISA, HI and VNT. The box and whisker plots indicate the minimum, maximum, median, and 25% and 75% quartiles. Each dot represents an individual result. For the ELISA and HI plots, the grey area delimits the threshold for positive results (S/N < 0.6 for ELISA) or a predicted correlate of protection (1:40 in HI). The number over the box indicates the proportion of examined individuals over the threshold, with the number in red corresponding to the proportion of seropositive animals at the moment when swIAV incidence was the highest for that batch of pigs. For VNT, no threshold is indicated, as no standard threshold has been determined for pigs. The average S/N values or titres were compared between consecutive weeks and are indicated in the graphs. *p < 0.05; **p < 0.01; ***p < 0.001; ****p < 0.0001; n.s. = nonsignificant difference.

In F2, MDA was present in most of the animals at the 1^st^ woa of L1 and L2, as shown both by ELISA and HI. In L1, the proportion of seropositive animals sharply decreased at 3 woa, which coincided with the peak incidence. By 8 woa, only 24.3% of the animals were positive by ELISA, and 56.3% of the animals produced HI titres ≥ 5.3 log_2_. In L2, a sharp decrease in the proportion of HI seropositive pigs was observed at 4 woa, which coincided with the peak incidence of swIAV. By 8 woa, only 18% of the piglets had HI titres ≥ 5.3 log_2_ (35% positive by ELISA), a significantly lower value than that of L1 piglets (*p* < 0.05). In this case, the proportion of HI-positive pigs at 12 woa significantly increased to 68% (*p* < 0.05). The VNT results in L1 tended to decrease gradually over time, whereas in L2, the titres remained more constant (Figure 3).

**Figure 4:**
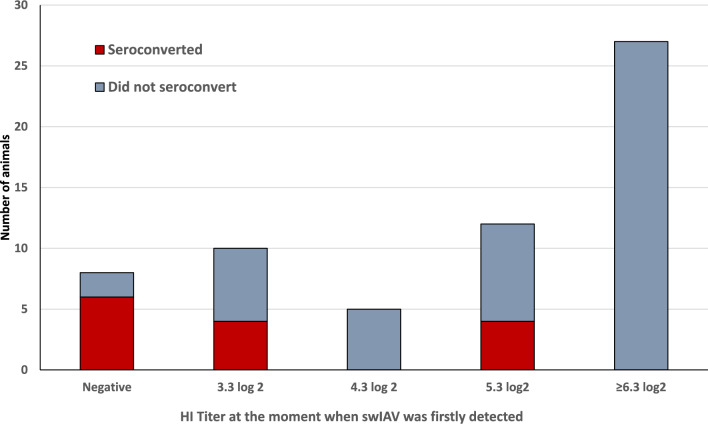
**Seroconversion and HI titre when animals were infected by swIAV for the first time.** The graph shows the number of animals for which an increase in the HI titre ≥ X2 was observed after infection with respect to the HI titre determined when infected by swIAV for the first time. The animals were examined 2–4 weeks after infection, depending on the periodicity of sampling.

We then examined whether the HI titre at the initial swIAV detection could be linked to seroconversion 15 days later (or 4 weeks in the case of animals infected at 8 woa in L2). Titres ≥ 6.3 log_2_ were clearly associated with a lack of seroconversion (*p* = 0.001). Thus, while 40% of the examined pigs (14/35) seroconverted if infected when HI titres were lower than 6.3 log_2_, none of the 27 infected pigs had an HI titre ≥ 6.3 log_2_. Figure 4 shows the detailed distribution by titre. However, notably, many animals with low HI titres, or even some that tested negative when infected, did not seroconvert. C_t_ values were not related (R^2^ = 0.01, *p* = 0.493) to the HI titres at the time when pigs were initially detected as RT‒qPCR positive.

### Characterization of swIAV prolonged shedders and pigs that experienced reinfections

**Figure 5:**
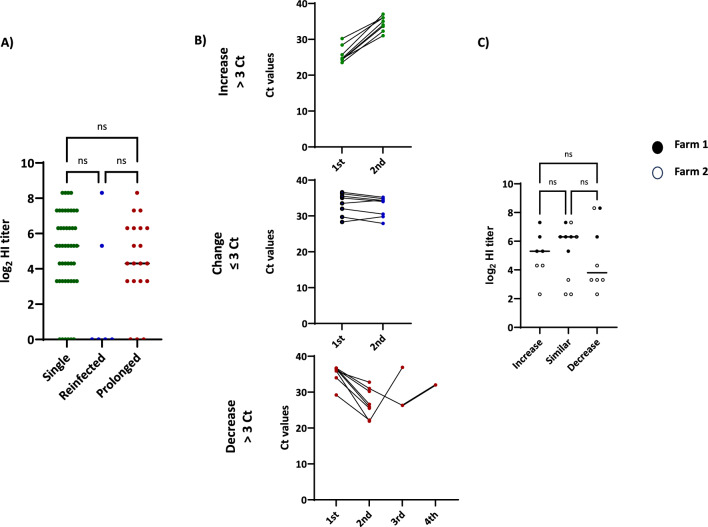
**Characterization of prolonged shedders and pigs reinfected with swIAV.** **A** The graph shows the distribution of HI titres at the moment when the first swIAV infection was detected in single shedders, prolonged shedders, and reinfected pigs. **B** Variation in C_t_ values between the first and consecutive detections of swIAV in nasal swabs from prolonged shedders. Upper: C_t_ values for cases in which the second detection resulted in a decrease in the viral load higher than 3.3 C_t_ units; middle: C_t_ values for animals that showed similar viral loads in consecutive samplings; and lower: C_t_ values for cases where viral loads increased between consecutive samples. **C** Distribution of HI titres when swIAV was detected for the first time in the animals shown in **B**.

Next, we aimed to characterize the animals that exhibited prolonged swIAV shedding or reinfection. Since previous studies suggested that prolonged shedding might be the result of interference with MDA, we first examined whether this phenomenon occurred in our case. Figure 5 summarizes the results. When the HI titres of the animals that were first detected as infected in prolonged shedders were compared with those of single shedders or with those of the animals that experienced reinfections, no differences were detected. In fact, shedding for one week or more could be observed within the whole range of HI titres, from negative to 8.3 log_2_ (negative-1:320).

We then examined the pattern of shedding. The set of prolonged shedders was classified on the basis of whether the C_t_ values increased (indicating a decreasing viral load), decreased (indicating increasing viral loads), or remained similar between different positive samples. A change was considered significant if C_t_ values increased or decreased by more than 3.3 between samplings (equivalent to a decrease or increase in the viral load of 1 log_10_). However, no significant relationship was observed between the HI titre at the time of the first viral detection and the pattern of shedding.

Since HIs detect both neutralizing and non-neutralizing antibodies, we then examined the involvement of neutralizing antibodies alone using the VNT. Differences in the VNT titres at the time of initial detection of swIAV in each animal were not significant between single and prolonged shedders (8.1 ± 1.4 vs. 7.3 ± 1.7 log_2_).

In total, 6 animals were identified as reinfected by swIAV after testing negative at least one week before. At the time of the second detection, 5 patients were HI negative, while the sixth had an HI titre of 5.3 log_2_. Upon examination of the sera of those animals by VNT, titres ranged between negative and 5.3 log_2_ for the animals that tested negative in the HI and 9.3 log_2_ for the animals that tested positive.

Next, we examined whether PRRSV infection established before or at the time of swIAV infection could be related to prolonged shedding or a second infection by the same swIAV. Among the animals that tested positive only once for swIAV, 10.5% were coinfected with PRRSV (6/57). However, among those showing repeated swIAV infection, 36.6% were coinfected by PRRSV when the first infection occurred (6/16), whereas of those who tested positive for swIAV in two consecutive samplings or more, 50% were coinfected by PRRSV (13/26, Fisher’s exact *p* < 0.001). When considering only single shedders versus animals with altered shedding patterns (repeaters + prolonged shedders), animals that were PRRSV positive when infected by swIAV were 4 times more likely to have an altered shedding pattern than PRRSV-negative animals were (relative risk: 4.1, CI_95%_: 1.9–9.4).

## Discussion

Published evidence clearly indicates that swIAV is widespread in intensive pig production farms, where it tends to become endemic [10, 12, 37–39]. The persistence of infection on farms is an ideal scenario to facilitate the occurrence of reassortment events and the emergence of escape mutants. In addition, the sustained circulation of swIAV within the herd may lead to recurrent episodes of respiratory disease in nurseries or fattening units. Most often, many other agents co-circulate on farms, among which PRRSV is considered a major cause of respiratory disease. The interplay between these co-circulating pathogens greatly influences eventual clinical outcomes (reviewed by Saade et al. [23]).

The initial aim of the present study was to assess the dynamics of swIAV infection in endemic farms and to relate them to potential driving factors. However, during the course of the study, both farms examined became infected by a highly virulent PRRSV strain from a new clade, which was introduced in Spain in 2020 [25, 40]. The initial sampling was scheduled to detect circulation in the farrowing units and nurseries with a 2–3-week periodicity. However, the introduction of the new PRRSV strain significantly impacted the farm records, prompting us to modify the sampling strategy. We transitioned to a more intense sampling approach, conducting weekly or biweekly sampling and extending the sampling to fattening units (located offsite). This adjustment allowed for a more thorough examination of the evolution of swIAV and PRRSV infections.

The introduction of the new PRRSV strain coincided with a notable shift in the dynamics of swIAV infection within both farms. In F1, the cumulative incidence increased from 20% to 67.5%, together with the appearance of animals exhibiting prolonged viral shedding for 1 week or more. Similarly, in F2, the cumulative incidence rose from 50 to 70%, but more importantly, a large proportion of infected pigs showed extended shedding or presented reinfections after testing negative for swIAV for one or more weeks. Notably, both pre- and post-PRRSV introduction, some swIAV-positive samples yielded high C_t_ values that could represent active but low shedding but could also be the result of environmental contamination. However, when we compared the proportion of high C_t_ values (> 30) before and after the introduction of highly virulent PRRSV, no differences were found (not shown), indicating that this was not a factor involved in the observed changes.

The analysis of the animals that tested positive for swIAV on multiple occasions revealed different patterns. While animals exhibiting a clear decrease or increase in viral loads between the first and second detections could be attributed to sampling at the beginning and at the end of the shedding period, the behavior of those with relatively constant viral loads, as determined by C_t_ values, was more perplexing. These animals were not significantly different from the other animals in terms of their MDA titres or seroconversion patterns (data not shown). Nonetheless, these findings reveal that, under the conditions of the examined farms, the shedding period of swIAV was longer than one week for a substantial proportion of pigs.

In previous works, unusual shedding patterns have been related to MDA interference [17, 20, 21]. In our study, the fact that a specific animal was positive for swIAV on more than one occasion could not be related to the MDA titres at the time of infection but was related to PRRSV infection concurrent with the initial swIAV infection. In our opinion, both mechanisms are possible. For example, in F1L1, one animal experienced reinfection by the same swIAV, despite the farm being PRRSV negative at that time. Most likely, several factors can lead to the same situation. The profound impact of PRRSV on the immune system is well documented, with highly virulent strains capable of inducing strong apoptosis in the thymus and modulating T-cell responses (reviewed by Wang et al. [41]).

Establishing an experimental model of coinfection in animals with or without swIAV-specific MDA would help to clarify this issue. Notably, in three instances where an animal already infected by PRRSV was subsequently infected by swIAV, PRRSV viremia (but not nasal shedding) became undetectable for one or more weeks. This observation is consistent with previous reports [42], suggesting that swIAV may interfere with PRRSV infection because of the induction of high levels of interferon-α.

In our study, the levels of MDA were related to seroconversion, as none of the animals with MDA HI titres ≥ 6.3 log_2_ were infected by swIAV seroconversion. This finding could explain the declining trend of seropositivity observed over time in L1 of F1 and F2. In contrast, in L2 of both farms, the proportion of seropositive animals remained more constant, likely reflecting greater viral circulation and increased virus exposure. In any case, for the infected animals, the antibody titre at the time of infection did not seem to affect the viral load, since the C_t_ values were similar for all animals. This observation agrees with the findings of Deblanc et al. [18], further reinforcing the notion that MDA may contribute to the persistence of swIAV infections in pig herds. Notably, however, a lack of seroconversion was also observed in animals with lower HI titres. One potential explanation could be the presence in the MDA of antibodies that could block seroconversion but that were not detected in the HI tests. Since no other swIAV was found circulating on the farm, the origin of those blocking antibodies should be another H1 strain that infected sows in the past. Unfortunately, it is very difficult to guess which H1 clade could have resulted in those cross-reacting antibodies. One option could have been testing sera against a representative panel of H1 swIAV strains. However, this was beyond the scope of the present study, and the amount of collected sera from these very young animals was not enough to do that after all previous testing.

Certainly, one factor that could have also influenced the change in the pattern of detection of swIAv is the introduction of new swIAV groups or variants. Diaz et al. [43] reported that some herds may be infected by different groups of swIAVs and their variants that can coexist in what was called “genomic constellations”. In our case, sequencing revealed that, within each farm, all sequenced isolates belonged to the same group and were closely related, with amino acid similarities > 99.5% for the HA gene. However, we cannot fully discard the low-level circulation of other swIAV. In any case, if that occurred, the incidence of those other strains should have been very low to have a major impact on the observed circulation patterns.

Another interesting observation from the present study was the duration of PRRSV nasal shedding. On average, pigs infected by the highly virulent strain circulating in both farms during L2, particularly in F2, exhibited shedding periods exceeding 1 month, with some animals (e.g., n°83 in F2 L2) shedding for up to 12 weeks. These values surpass those reported for most PRRSV-1 isolates [44], which aligns with the notion that PRRSV strains with enhanced virulence display increased replication in the nasal mucosa [45].

Mortality rates were also very high at both farms following the introduction of the new PRRSV strain. Mortalities up to 12 Woa dramatically reached 32.5% in F1 and 55% in F2. In F1, mortality occurred after the peak incidence of PRRSV at 7 woa, whereas in F2, mortality was clearly related to the age at which piglets were infected by PRRSV (83% of the piglets born to the piglets with PRRSV viremia died). These findings clearly suggested that the introduction of highly virulent PRRSV was the main cause of death and that its impact was greater if infections occurred at earlier ages. Similarly, in F2, neonatal PRRSV infections precipitated respiratory disease in piglets as young as 1 woa. These results underscore the profound impact of the introduction of the highly virulent PRRSV strain on both farms.

## Supplementary Information

**Additional file 1. Individual results of RT‒qPCR analysis of nasal swabs for the detection of swIAV, PRRSV viremia, and PRRSV nasal shedding.**

**Additional file 2. Distribution of Ct values for swIAV-positive samples from Farms 1 (left) and 2 (right) for longitudinal follow-ups 1 (L1) and 2 (L2)**.

## Data Availability

Publicly available datasets were analysed in this study. Data will be shared upon reasonable request. The swIAV sequences are available at GenBank under the accession numbers PP457373-PP451380, PP440238-PP440245, PP439811-PP439818, and PP457394-PP457401.

## Abbreviations

IAV: influenza A virus
PRDC: porcine respiratory disease complex
swIAV: swine influenza A virus
MDA: maternally derived antibodies
PRRSV: porcine reproductive and respiratory syndrome virus
HI: hemagglutination inhibition test
VNT: viral neutralization test
PCV2: porcine circovirus type 2
woa: week of age
L1: longitudinal study 1
L2: longitudinal study 2
S: sneezing
C: coughs
DC: deep coughs
MDCK: Madin–Darby canine kidney cells
PAM: porcine alveolar macrophages
OD: optical density
