# Supplementary figures and images for: The introduction of a highly virulent PRRSV strain in pig farms is associated with a change in the pattern of influenza A virus infection in nurseries
Source: Vet Res. 2024 Nov 9;55:147. doi: 10.1186/s13567-024-01406-7 (PMC11549838; doi:10.1186/s13567-024-01406-7)

## Slide 1
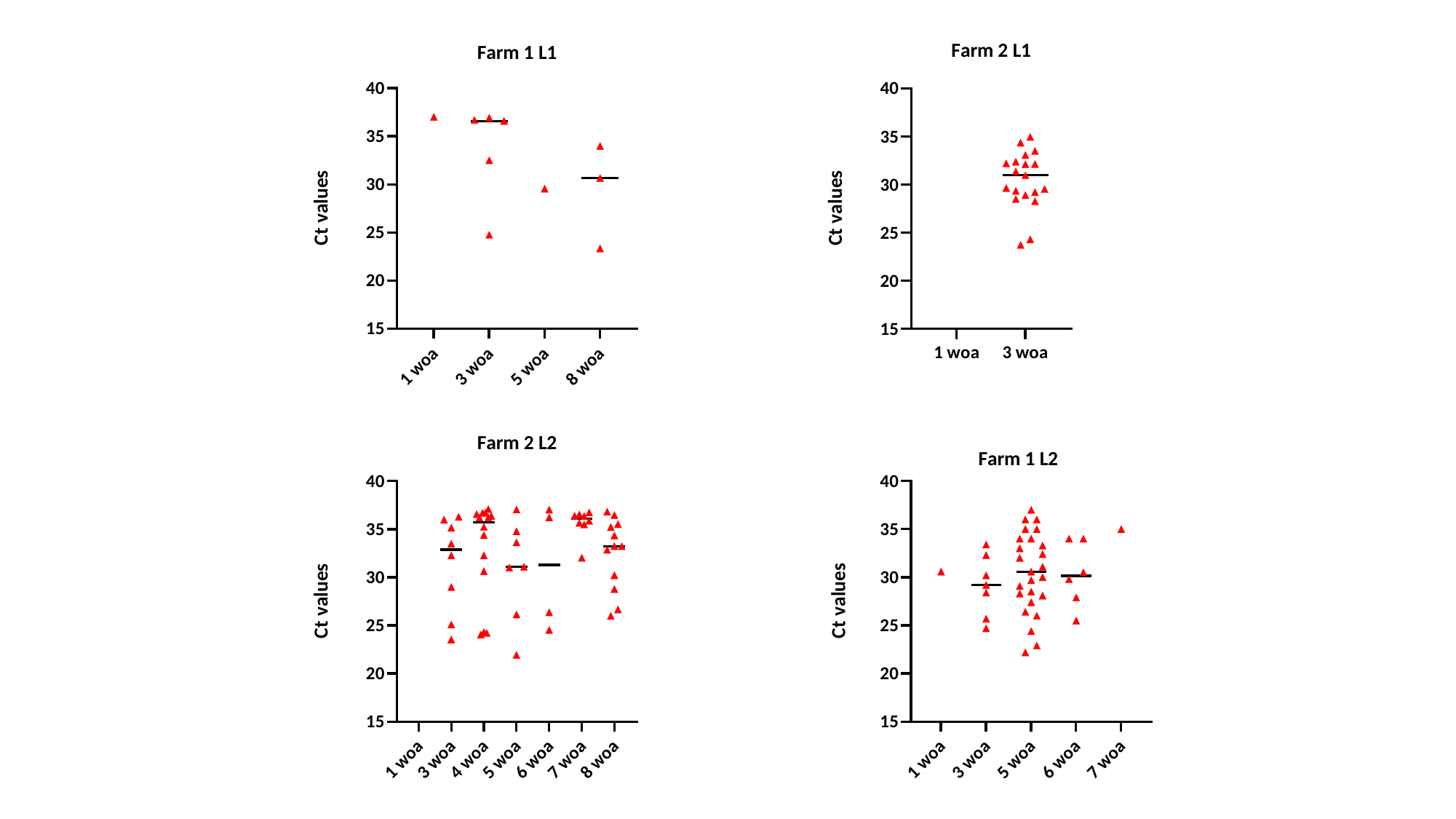

Supplement: Supplementary file 2 — Additional file 2. Distribution of Ct values for swIAV-positive samples from Farms 1 (left) and 2 (right) for longitudinal follow-ups 1 (L1) and 2 (L2). [file 13567_2024_1406_MOESM2_ESM.pptx]
